# Modelling Hollow Microneedle-Mediated Drug Delivery in Skin Considering Drug Binding

**DOI:** 10.3390/pharmaceutics17010105

**Published:** 2025-01-14

**Authors:** Tanmoy Bhuimali, Sarifuddin, Diganta Bhusan Das, Prashanta Kumar Mandal

**Affiliations:** 1 Department of Mathematics, Visva-Bharati University, Santiniketan 731235, WB, India; tanmoybhuimali@gmail.com; 2 Department of Mathematics, Berhampore College, Berhampore 742101, WB, India; suind1213@gmail.com; 3 Chemical Engineering Department, Loughborough University, Loughborough LE11 3TU, Leicestershire, UK

**Keywords:** hollow microneedle, injection velocity, drug binding, aspect ratio, viscoelastic skin, interstitial fluid

## Abstract

**Background/Objectives:** Microneedle(MN)-based drug delivery is one of the potential approaches to overcome the limitations of oral and hypodermic needle delivery. An in silico model has been developed for hollow microneedle (HMN)-based drug delivery in the skin and its subsequent absorption in the blood and tissue compartments in the presence of interstitial flow. The drug’s reversible specific saturable binding to its receptors and the kinetics of reversible absorption across the blood and tissue compartments have been taken into account. **Methods:** The governing equations representing the flow of interstitial fluid, the transport of verapamil in the viable skin and the concentrations in the blood and tissue compartments are solved using combined Marker and Cell and Immersed Boundary Methods to gain a quantitative understanding of the model under consideration. **Results:** The viscoelastic skin is predicted to impede the transport of verapamil in the viable skin and, hence, reduce the concentrations of all forms in the blood and the tissue compartments. The findings reveal that a higher mean concentration in the viable skin is not always associated with a longer MN length. Simulations also predict that the concentrations of verapamil in the blood and bound verapamil in the tissue compartment rise with decreasing tip diameters. In contrast, the concentration of free verapamil in the tissue increases with increasing injection velocities. **Conclusions:** The novelty of this study includes verapamil metabolism in two-dimensional viscoelastic irregular viable skin and the nonlinear, specific, saturable, and reversible binding of verapamil in the tissue compartment. The tip diameter and the drug’s injection velocity are thought to serve as regulatory parameters for the effectiveness and efficacy of MN-mediated therapy if the MN is robust enough to sustain the force needed to penetrate a wider tip into the skin.

## 1. Introduction

Over the last few decades, transdermal drug delivery (TDD) research has concentrated on creating new techniques, including iontophoresis, sonophoresis, chemical permeation enhancers, and microneedles (MNs), to improve and control drug permeation and skin penetration. Many noteworthy therapeutic benefits, including fewer side effects, controlled dosage, and increased patient compliance, are provided by TDD [1,2]. Although taking pharmaceuticals orally is an easy option, not all drugs can be taken this way to the targeted site of action. In the cases of TDD, injecting a drug with a hypodermic needle is an acceptable alternative. However, this kind of needle-based delivery presents issues for patients because it can result pain at the administration site. Because of the skin’s protective, inflammatory, and immunological properties, the MN-based TDD system offers an enticing alternative to overcome the limitations of previous approaches [3,4,5,6,7,8,9]. The strong barrier qualities of the stratum corneum (SC) have restricted the application of TDD. However, it has numerous benefits over alternative delivery methods.

Minimally-invasive MN arrays, consisting of micron-scale needles, have been developing since the late 1990s. Each array is made up of tiny needles that stick out from a support that resembles a patch. These needles may be based on polymer, glass, metal, or silicon [10,11]. Various drugs can be carefully injected into tissue using the MNs. Their insertion does not produce pain, since their shorter lengths prevent them from penetrating the nerve system [12,13,14]. These devices can be used for biomarker extraction and the systemic and localised delivery of drugs, vaccinations, and other biologics. These devices are presently being investigated as potential methods of delivering the COVID-19 vaccination as well [15,16,17,18,19]. Concomitantly, MNs have been used to study the delivery of a variety of substances, such as peptides, cancer drugs, and opioids [20,21].

A number of varieties of MNs have been fabricated for both research and commercial purposes, namely, solid, hollow, coated, biodegradable/dissolving, and hydrogel-forming MNs [12,22,23,24,25]. To evaluate the blood concentration and skin penetration of drugs administered by MN arrays, various mathematical models have been developed [8,9]. In several preclinical studies and small-scale clinical trials, MNs have demonstrated potential in the transdermal administration of molecules, desmopressin, DNA, vaccines, insulin, and human growth hormones [26,27]. Additionally, a great deal of research has been conducted on MNs for blood sampling, biosensing key biological molecules, and gene transfer with the help of MNs [28,29,30].

Given that the hollow MNs (HMNs) share a basic structure with a standard hypodermic needle (a lumen through which a therapeutic substance flows), they have the potential to overcome the dosage limitation associated with solid MNs by delivering relatively large amounts of drugs into the skin [31,32]. HMNs are designed to continuously distribute drugs through the needle’s bore by penetrating the SC (10–15 μm) and reaching the viable skin (VS) because of this shared structure [31]. Through diffusion, pressure, or electrical processes, HMNs provide the advantages of flow and dose regulation in lab-on-a-chip systems [33]. Vaccines, proteins, mRNA, and diagnostic supplies can all be delivered via HMNs. Pharmacologically active insulin has been demonstrated to be delivered by HMNs to diabetic rats [30,34]. Many interrelated physiological and physicochemical systems are involved in the administration of drugs. Investigating every process through in vivo research would be unfeasible or uneconomical. An alternative method is provided by mathematical modelling. A set of validated governing equations for the drug delivery processes is used to characterise the processes [35,36]. HMNs have been employed in the development of mathematical frameworks for drug delivery transdermally. As the needle-inserted drug is metabolised while passing through the VS [37,38,39,40,41], the inclusion of metabolism into the model studied is essential [37,41,42,43]. Collagen, elastic fibres, and proteoglycans are the primary factors influencing the skin’s mechanical properties, making it a viscoelastic tissue [44,45,46]. It has been established that drug transport in viscoelastic materials, like skin, deviates from the standard Fick’s rule and should be described in terms of two significant contributions to the total verapamil transport of Fickian ($J_{F}$) and non-Fickian ($J_{nF}$) fluxes [47,48,49,50,51,52,53,54]. The expressions for $J_{F}$ and $J_{nF}$ are given in the next section.

The present study concerns a two-compartment body model with a monolayer viscoelastic skin in which verapamil metabolism occurs in VS. Verapamil is utilised for various medical applications, including the prevention of myocardial infarction [55], reduction of hypertension [56], cessation of cluster headache episodes [57], and management of diarrhoea associated with microscopic colitis [58]. The oral bioavailability of verapamil may decrease by 80% to 90% [59]. Conversely, others contend that it can be administered via the transdermal method to enhance bioavailability [42]. Verapamil is well recognised to bind to α-adrenergic receptors on a range of tissues, such as the heart, cardiac sarcolemmal membranes, rat kidneys, and human platelets [60,61]. This binding is saturable and reversible. Very recently, verapamil transport in the viable skin and its subsequent binding in the tissue have been successfully investigated, both analytically and numerically, in which the release of verapamil from an MN tip was considered as a Dirichlet condition [62]. Several factors, like the metabolism of verapamil in VS, the skin’s viscoelastic nature, the interstitial flow in VS, water loss to the atmosphere, verapamil removal by the lymphatic system, average injection velocity, MN tip diameter, and the nonlinear specific saturable and reversible binding of verapamil in the tissue compartment, are included in the model considered. This study’s novel aspects include verapamil metabolism in two-dimensional viscoelastic irregular VS and the nonlinear, specific, saturable, and reversible binding of verapamil in the tissue compartment in contrast to previous studies on transdermal delivery using MN arrays. Because the skin is a viscoelastic material, both Fickian and non-Fickian diffusion for the transport of verapamil in the skin have been paid adequate attention [53]. The governing equations representing the metabolised transport of verapamil in the two-dimensional viscoelastic irregular domain, the blood (plasma) concentration, and the concentrations (free and bound) of verapamil in the tissue compartment are solved numerically by leveraging the Marker and Cell (MAC) method [63,64] and the Immersed Boundary Method (IBM) [65,66,67]. The unique aspect of this investigation is how the interstitial velocity and pressure are perturbed due to MN-based verapamil delivery. This investigation cannot rule out the effects of the aspect ratio (AR), tip diameter, and average injection velocity of verapamil on the transient concentrations. As far as the authors are aware, no such studies have considered the binding of verapamil in the tissue compartment when using MN for verapamil delivery in the skin.

## 2. Material and Methods

### 2.1. Model Assumptions

**Figure 1:**
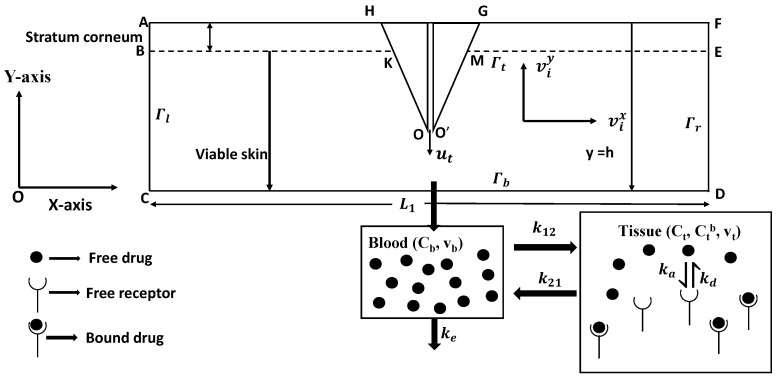
Schematic of transdermal verapamil delivery from MN array.

While studying HMN-based transdermal verapamil delivery and its subsequent binding with the specific receptors, the following assumptions are made:

- The VS is considered a viscoelastic material [46], and the strain caused by the penetrant induces a viscoelastic stress response.
- Verapamil transport in viscoelastic material, like skin, deviates from the classical Fick’s law, and hence, the total verapamil transport consists of both Fickian and non-Fickian fluxes [54].
- The skin metabolism occurs in VS with first-order reaction kinetics because of lower in vivo drug concentrations, which are usually below the Michaelis–Menton (Monod) constant [37,68,69].
- Since the diffusion coefficient of verapamil in SC is three orders of magnitude less in SC as compared to the VS, the back diffusion of verapamil at the VS–SC interface (B−K, M−E) (cf. Figure 1) is disregarded [8,42,70].
- The effective diffusivity of verapamil in VS is assumed to be constant, as the diffusion coefficients in the epidermis and dermis are of the same magnitude [71], and the transport of verapamil in VS is considered to be two-dimensional.
- Instead of withdrawing the MN array after application duration, the cessation of verapamil delivery from the HMN is assumed, which disregards the consideration of the annular gap width. The annular gap width is prominent in the case of drug delivery from polymeric MN.
- Apart from monolayer skin, the body is assumed to have two compartments: the blood or plasma and the tissue. The blood compartment absorbs all unmetabolised verapamil molecules from the VS, and there is a reversible uptake between the blood and the tissue compartments, with first-order elimination kinetics from the blood compartment [8,22,37,72,73].
- A nonlinear, specific, saturable and reversible binding of verapamil with its receptors in the tissue compartment has been taken into account [42,60,61].

Figure 1 is a schematic diagram of the two-dimensional mathematical framework created for modelling the delivery of verapamil through the skin using an HMN with a drug reservoir. The uppermost layer of skin, known as the SC, serves as the primary barrier to verapamil delivery [74,75,76].

### 2.2. Formulation of the Problem

#### 2.2.1. Flow of Interstitial Fluid in VS

Considering the VS tissue as a biological porous medium with measurable porosity and permeability, and since interstitial fluid is Newtonian and has low velocity through the skin, the governing equations in the steady state condition can be described by the continuity equation and Darcy’s law as [77,78]

(1) $$\begin{array}{ccc} \nabla \cdot v_{i} & = & F_{bl}-F_{ly}, \end{array}$$

(2) $$\begin{array}{ccc} v_{i} & = & -\frac{k}{\mu_{f}}\nabla p, \end{array}$$

where *k* is the Darcy permeability of the interstitium in units of m^2^, $\mu_{f}$ is the viscosity, *p* is the pressure, and $v_{i}$(=$v_{i}^{x}$, $v_{i}^{y}$) is the interstitial flow velocity vector.

Starling’s law governs the fluid loss from the blood as [79]

(3) $$\begin{array}{c} F_{bl}\;=\;L_{bl}\frac{S_{bl}}{V_{tis}}\left[p_{bl}-p-\sigma_{T}\left(\pi_{bl}-\pi_{i}\right)\right], \end{array}$$

where $L_{bl}$ is the hydraulic conductivity of the microvasculature wall, $\frac{S_{bl}}{V_{tis}}$ stands for the microvasculature density—which is defined as the surface area of the microvasculature wall per tissue volume—$p_{bl}$ is the pressure of blood, $\sigma_{T}$ is the osmotic reflection coefficient due to the proteins in the blood, and $\pi_{bl}$ and $\pi_{i}$ are the osmotic pressure of the blood and interstitial fluid, respectively.

The fluid loss to the lymphatic system in the skin can be calculated as [80]

(4) $$\begin{array}{c} F_{ly}\;=\;L_{ly}\frac{S_{ly}}{V_{tis}}\left(p-p_{ly}\right), \end{array}$$

where $L_{ly}$ is the hydraulic conductivity of the lymphatic vessel wall, $\frac{S_{ly}}{V_{tis}}$ is the surface area of the lymphatic vessel wall in unit tissue volume, and $p_{ly}$ is the lymphatic pressure.

#### 2.2.2. Boundary Conditions for ISF in VS

Since a proper boundary condition for ISF at the VE–SC interface is apparent as it varies at different states, namely, at equilibrium and dynamic states, some researchers opined that the velocity of ISF is zero at the VE–SC interface, considering the impermeable nature of SC [81]. In a seminal work [82], the transdermal water loss (TEWL) from inside the body at the steady state was predicted by considering that the influx of water into the SC from VE and the efflux of water from the SC to the surrounding atmosphere are equal. They calculated the evaporation of the water loss rate from the SC to the surrounding atmosphere ($f_{b}$) using

$$\begin{array}{c} f_{b}=k_{g}\frac{\left(a_{w}-RH\right)p_{sat}^{0}MW}{RT}, \end{array}$$

where $p_{sat}^{0},$ the water saturated vapour pressure at the SC surface; T, is the temperature; MW is the water molecular weight; R, is the gas constant; $k_{g},$ is the mass transfer rate; RH, is the ambient relative humidity, and $a_{w}$ is the SC surface water activity. At dynamic equilibrium, $a_{w}∼RH$ and the dependence of TEWL can be expressed by an empirical formula as [82]

$$\begin{array}{ccc} f_{TEWL} & = & -2.25\exp\left(\frac{RH}{3.18}\right)-2.97\times 10^{-3}\exp\left(\frac{RH}{1.34\times 10^{-1}}\right)\\ & - & 1.41\times 10^{-15}\exp\left(\frac{RH}{2.79\times 10^{-2}}\right)\\ & + & 1.64\times 10\;\;\;\;\;(g/m^{2}/hr), \end{array}$$

at VE-SC interface; B-K, M-E (cf. Figure 1).

In another study on MN-mediated TDD [78], the VE–SC interface condition is based on the flux of TEWL at a dynamic equilibrium state. In the line of [78], the VE–SC interface condition may be assumed as

(5) $$\begin{array}{ccc} ISF_{flux} & = & f_{TEWL}. \end{array}$$

At the skin–MN interface ($K-O,\;O^{\prime }-M$) in the schematic diagram, the ISF velocity is assumed to be the usual no-slip. The ISF flux at the bottom of the VS (y=0, C−D) is considered to be zero. At the boundaries [$B-C\;\left(x=0\right),\;D-E\left(x\;=\;L_{1}\right)$], no-flux conditions are specified. During the duration of application of the MN array $\left(0\leq t\leq t_{a}\right)$, the velocity components in the *x* and *y* directions at the tip of the MN ($y=h-L,\;O-O^{\prime }$) are as follows: $v_{i}^{x}$ = 0, and $v_{i}^{y}$ = $-u_{t}$, where $u_{t}$ is the average injection velocity of verapamil for a single MN at the MN tip in a direction opposite to $v_{i}^{y}$, which, after the application duration ($t>t_{a})$, should be read as $v_{i}^{x}\;=\;0\;=\;v_{i}^{y}$ (cf. Figure 1).

### 2.3. Viscoelastic Behaviour of VS

The Boltzmann integral describes the viscoelastic behaviour of the skin by [54]

(6) $$\begin{array}{c} \sigma \left(x,y,t\right)\;=\;-\int_{0}^{t}E\left(t-s\right)\frac{\partial \epsilon (x,y,s)}{\partial s}ds, \end{array}$$

where *E* represents the Young modulus of the skin, and the strain ϵ caused by the penetrant induces a viscoelastic stress (σ) response with the opposite sign.

Considering that the strain is linearly related to the concentration of verapamil [54,83], we may assume

(7) $$\begin{array}{c} \epsilon (x,y,t)\;=\;\alpha C(x,y,t), \end{array}$$

where α (mL/μg) is the proportionality constant between the strain and the concentration. Using (7) in (6), we obtain

(8) $$\begin{array}{c} \sigma \left(x,y,t\right)\;=\;-\alpha \int_{0}^{t}E\left(t-s\right)\frac{\partial C(x,y,s)}{\partial s}ds. \end{array}$$

The Maxwell–Wiechert model gives the Young modulus of the skin as a combination of the Young modulus of spring and Maxwell fluid (spring–dashpot model for the viscoelastic material):

(9) $$\begin{array}{c} E\left(s\right)\;=\;E_{0}+E_{1}e^{-\frac{s}{\tau }}, \end{array}$$

where $E_{0}$ is the Young modulus of the free spring, and $E_{1}$ is the Young modulus of the Maxwell fluid. Here, the relaxation time τ is given by

(10) $$\begin{array}{c} \tau \;=\;\frac{\mu }{E_{1}}, \end{array}$$

where μ is the viscosity of a Maxwell fluid.

Using the Equations (9) and (10) in Equation (8), we have

(11) $$\begin{array}{c} \sigma \left(x,y,t\right)\;=\;-\alpha \left(E_{0}+E_{1}\right)C\left(x,y,t\right)+\alpha \left(E_{0}+E_{1}e^{-\frac{t}{\tau }}\right)C\left(x,y,0\right)+\alpha \frac{E_{1}}{\tau }\int_{0}^{t}e^{-\frac{\left(t-s\right)}{\tau }}C\left(x,y,s\right)ds. \end{array}$$

### 2.4. Governing Equations in VS

Solute transport properties in viscoelastic VS are known to defy the classical Fick’s law in drug delivery. Before verapamil is absorbed in the blood compartment, its transport can be expressed as follows to account for the skin’s viscoelastic behaviour:

(12) $$\begin{array}{c} \frac{\partial C}{\partial t}+\nabla \cdot \left(v_{i}\;C\right)\;=\;-\nabla \cdot J-k_{m}C, \end{array}$$

where *C* is the volume-averaged verapamil concentration in VS, $k_{m}$ is the first-order metabolic reaction rate constant, and *t* is the time. Here, the total flux (*J*) can be written as $J\;=\;J_{F}+J_{NF}$, $J_{F}$ is the Fickian flux and $J_{NF}$ is the non-Fickian flux, which are defined by

(13) $$\begin{array}{c} \begin{array}{ccc} J_{F} & = & -D_{vs}^{e}\nabla C,\\ J_{NF} & = & -D_{v}\nabla \sigma , \end{array} \end{array}$$

where $D_{vs}^{e}$ represents the effective diffusion coefficient in the VS, which is defined as

(14) $$\begin{array}{ccc} D_{vs}^{e} & = & \frac{\phi }{\tau_{s}}D_{vs}, \end{array}$$

$D_{vs}$ is the diffusion coefficient of verapamil in the skin; $\tau_{s}$ is the tortuosity; and $D_{v}$ is the stress-driven coefficient.

Using the Equation (13) in (12), we obtain

(15) $$\begin{array}{c} \frac{\partial C}{\partial t}+\nabla \cdot \left(v_{i}\;C\right)=\nabla \cdot \left(D_{vs}^{e}\nabla C+D_{v}\nabla \sigma \right)-k_{m}C. \end{array}$$

Now,

(16) $$\begin{array}{ccc} J_{NF} & = & -D_{v}\nabla \sigma \left(x,y,t\right)\\ & = & -D_{v}\nabla \left(-\alpha \left(E_{0}+E_{1}\right)C\left(x,y,t\right)+\alpha \left(E_{0}+E_{1}e^{-\frac{t}{\tau }}\right)C\left(x,y,0\right)+\alpha \frac{E_{1}}{\tau }\int_{0}^{t}e^{-\frac{\left(t-s\right)}{\tau }}C\left(x,y,s\right)ds\right)\\ & = & \alpha \left(E_{0}+E_{1}\right)D_{v}\nabla C\left(x,y,t\right)-\alpha \frac{E_{1}}{\tau }D_{v}\int_{0}^{t}e^{-\frac{\left(t-s\right)}{\tau }}\nabla C\left(x,y,s\right)ds \end{array}$$

Putting this value in (15), finally we have

(17) $$\begin{array}{ccc} \frac{\partial C}{\partial t}+\nabla \cdot \left(v_{i}\;C\right) & = & D_{vs}^{e}\nabla \cdot \left(\nabla C\right)-\alpha \left(E_{0}+E_{1}\right)D_{v}\nabla \cdot \left(\nabla C\right)\\ & + & \alpha \frac{E_{1}}{\tau }D_{v}\int_{0}^{t}e^{-\frac{\left(t-s\right)}{\tau }}\nabla \cdot \left(\nabla C\left(x,y,s\right)\right)ds-k_{m}C \end{array}$$

### 2.5. Cumulative Amount of Verapamil Permeated

The rate of verapamil permeation across the skin can be calculated as

(18) $$\begin{array}{c} \frac{dQ}{dt}\;=\;\int_{0}^{L_{1}}{\left[-D_{vs}^{e}\left(\frac{\partial C}{\partial y}\right)-D_{v}\left(\frac{\partial \sigma }{\partial y}\right)\right]}_{y=h}\;dx. \end{array}$$

The cumulative amount of verapamil permeated per unit area is

(19) $$\begin{array}{c} Q=\int_{0}^{t}\left(\frac{dQ}{dt}\right)\;dt\;=\;-\int_{0}^{t}\int_{0}^{L_{1}}{\left[D_{vs}^{e}\left(\frac{\partial C}{\partial y}\right)+D_{v}\left(\frac{\partial \sigma }{\partial y}\right)\right]}_{y=h}\;dx\;\;dt. \end{array}$$

### 2.6. Governing Equations in the Blood and Tissue Compartments

The blood compartment absorbs all unmetabolised verapamil molecules in viscoelastic skin (Equation (20)), and there is a reversible uptake of verapamil between the tissue and blood compartments (Equation (21)). Free verapamil binds nonlinearly to the adrenergic receptors in the tissue compartment (Equation (22)):

(20) $$\begin{array}{ccc} v_{b}\frac{dC_{b}}{dt} & = & \left(\frac{dQ}{dt}\right)S-k_{e}C_{b}v_{b}-k_{12}C_{b}v_{b}+k_{21}C_{t}v_{t}, \end{array}$$

(21) $$\begin{array}{ccc} v_{t}\frac{dC_{t}}{dt} & = & k_{12}C_{b}v_{b}-k_{21}C_{t}v_{t}-k_{a}v_{t}C_{t}\left(B_{M}-C_{t}^{b}\right)+k_{d}C_{t}^{b}v_{t}, \end{array}$$

(22) $$\begin{array}{ccc} v_{t}\frac{dC_{t}^{b}}{dt} & = & k_{a}C_{t}v_{t}\left(B_{M}-C_{t}^{b}\right)-k_{d}C_{t}^{b}v_{t}, \end{array}$$

where $C_{b},\;C_{t},\;C_{t}^{b}$ are the concentrations in the blood compartment and the free and bound verapamil in the tissue compartment, respectively, and $B_{M}$ is the initial binding site concentration. $v_{b}$ and $v_{t}$ are the respective volumes of distribution in the blood and tissue compartments, $\frac{dQ}{dt}$ is the penetration rate of verapamil through the skin, *S* is the surface area of the MN array, $k_{e}$ is the elimination rate constant from the blood compartment into the systemic circulation, $k_{12}$ and $k_{21}$ are the transfer rate constants between the blood and tissue compartments, and $k_{a}$ and $k_{d}$ represent the association and dissociation rate constants in the tissue compartment.

### 2.7. Initial and Boundary Conditions for Verapamil

Initially, the concentration of verapamil in the two-dimensional VS domain except at the tip of the MN is assumed to be zero

(23) $$\begin{array}{c} C(x,y,0)=0\;\;\mathrm{at}\;\;t=0, \end{array}$$

The assumption of no flux of verapamil is given as (cf. Figure 1)

(24) $$\begin{array}{c} -D_{vs}^{e}\frac{\partial C}{\partial n}=0\;\mathrm{at}\;\mathrm{the}\;\mathrm{boundary},\;B-K,\;K-O,\;O^{\prime }-M,\;M-E,\;B-C,\;D-E,\;\forall \;t. \end{array}$$

At the tip of the MN (y=h−L, O-O′), verapamil is injected into the skin and can be expressed as

(25) $$\begin{array}{c} C_{flux}=\frac{\rho_{d}}{V_{skin}}\phi \;u_{t}S_{t}\;\;\;\;\mathrm{for}\;\;\;\;\;0\leq t\;\leq t_{a}, \end{array}$$

where $u_{t}\left(=\frac{u_{0}S}{nS_{t}}\right)$ is the average injection velocity of a single MN, $u_{0}$ is the effective injection velocity, $S_{t}\left(=\frac{\pi d_{tip}^{2}}{4}\right)$ is the cross-sectional area of a tip, $d_{tip}$ is the diameter of the MN tip, *n* is the number of needles in the MN array, ϕ is the porosity of the skin, $\rho_{d}$ is the density of verapamil in drug formulation, and $t_{a}$ is the duration of the application of the MN array.

At the bottom of the VS (y=0, C-D), a perfect sink condition is assumed as

(26) $$\begin{array}{c} C=0,\;\;y=h,0\leq x\leq L_{1},\;\;\forall \;t\;\left(\mathrm{CD}=L_{1}\right). \end{array}$$

Moreover, initially, there is no concentration in the tissue and blood compartments, that is,

(27) $$\begin{array}{c} C_{b}\;=\;C_{t}\;=\;C_{t}^{b}=0,\;\;\mathrm{at}\;t=0. \end{array}$$

### 2.8. Method of Solution 

For a detailed quantitative analysis of this model, the plausible values of input parameters are given in Table 1. We derive the pressure equation from the Equations (1) and (2) as

(28) $$\begin{array}{c} \nabla^{2}p=\frac{\mu_{f}}{k}\left(F_{ly}-F_{bl}\right), \end{array}$$

where $\nabla^{2}$ is the Laplacian operator.

The pressure Equation (28) is solved using the successive over-relaxation (S.O.R.) technique, with the overrelaxation parameter is set to 1.5. After having obtained the pressure for ISF in the VS from the Equation (28), we calculate the velocity components ($v_{i}^{x}$, $v_{i}^{y}$) from the Equation (2), and accordingly, the equations for verapamil concentrations (17, 20, 21, and 22) are solved numerically. The details of the numerical methods are appended below.

#### 2.8.1. MAC Methodology

**Figure 2:**
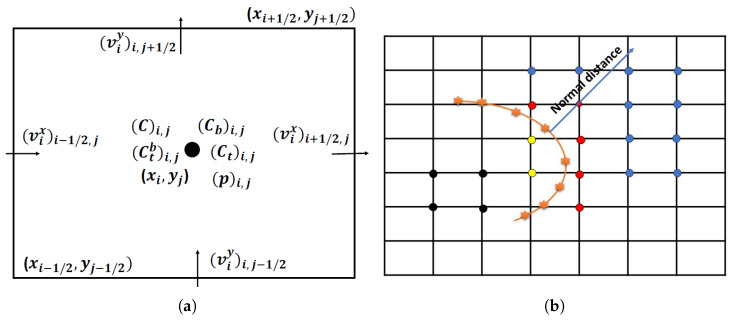
(**a**) A typical MAC cell in which the velocity components of ISF are located at the mid-points of its faces; the pressure for ISF and all concentrations ($C,\;C_{b},\;C_{t},\;C_{t}^{b}$) are placed at the cell centre. (**b**) Demarcation and interpolation of IBM nodes. IBM is used at the cut cells, where cells with blue dots are inside the domain of computation, and black dots are outside the computational domain. Points inside and outside the computational domain but close to the interface are identified as immersed points (red and yellow dots, respectively). Orange dots are on the actual boundary line.

A control volume-based finite-difference scheme in staggered grids is used to discretise the governing equations. In this grid alignment, the interstitial velocity components, the pressure, and verapamil concentrations are located at different positions (cf. Figure 2a). A first-order forward difference scheme to discretise time derivative terms, a combined second-order upwinding difference scheme for convective terms, and a second-order three-point central difference formula for diffusive terms have been put on record to discretise the equations.

#### 2.8.2. Immersed Boundary Method

The irregular domain boundaries in a Cartesian mesh’s background have been modelled using the immersed boundary approach. In the current implementation, a direct forcing strategy has been employed [65,66]. The immersed boundary approach is used at the intercepted cells to estimate the pressure, concentration, and velocity field values. By adding forcing terms to Darcy’s equation at grid points in intercepted cells—as seen in Figure 2b—a cell contains grid points identified as blue dots inside the fluid domain and black dots outside the fluid domain. Points inside and outside the fluid domain but close to the interface are identified as immersed points (red and yellow dots, respectively). Using a search strategy that computes the normal to the immersed surface from a nearby point and takes a dot product with the position vector of the nearest mesh point, the immersed points (red/yellow) are located.

Regarding the curved interface, the position of the immersed points is determined by the sign of the dot product. The value of the dot product of the surface normal vector $\vec{\eta }$ and the grid point position vector ($\vec{q}$) determines the grid point’s location in the fluid domain. The grid point is inside the fluid domain if $\vec{q}\cdot \vec{\eta }\geq 0$ and outside the fluid domain if $\vec{q}\cdot \vec{\eta }<0$. The IBM method aims to determine the unknown quantities at the boundary. To preserve the overall second-order accuracy of the solution algorithm, the following second-order polynomial function of the normal distance (η) from the immersed surface is taken into consideration: $f\left(\eta \right)\;=\;A\eta^{2}+B\eta +C$ is used to carry out the variables’ interpolation and extrapolation at the cut-cell grid locations [91,92,93], where η is the coordinate value along the surface normal direction. The unknown coefficients *A*, *B*, and *C* are found using known variables at three different places in the polynomial. Each immersed point has two along its standard path and one at the boundary where η = 0 is considered so that the value of *f* at the intercepted cell meets the necessary border criteria.

#### 2.8.3. Stability Criteria

For the two-dimensional convection reaction diffusion equation, the standard Courant–Friedrichs–Lewy (CFL) stability criterion, depending on the diffusivity and dimensions of the control volume, gives rise to the time step dt as [94]

(29) $$\begin{array}{c} dt\left(\frac{1}{dx^{2}}+\frac{1}{dy^{2}}\right)\max\left(D_{vs}^{e},D_{v}\right)<\frac{1}{2}, \end{array}$$

where (dx,dy) are the dimensions of the control volume along the *x* and *y* directions, respectively. This time step is used to determine the concentrations in the blood and tissue compartments.

## 3. Results

### 3.1. Grid Independence Study

**Figure 3:**
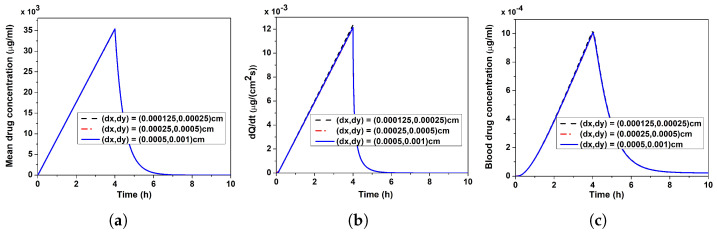
Grid independence study. (**a**) Mean verapamil concentration in VS. (**b**) Rate of verapamil permeation into blood compartment. (**c**) Blood concentration. Application duration ($t_{a}$) = 4 h.

Figure 3a–c displays a few simulations with varying step lengths (dx,dy) along the *x*- and *y*-directions (relatively coarser and finer grid sizes) for the mean verapamil concentration in VS, the rate of verapamil permeation into the blood compartment, and the blood concentration, respectively. These grid independence simulations were run in order to confirm the accuracy of the present numerical code and look into the error related to the grid sizes chosen. The fact that the mean concentration, permeation rate, and blood concentration exhibit near-overlapping transient behaviour with varying dx and dy amply illustrates the accuracy of the chosen grid sizes and found negligible impact.

### 3.2. Pressure, Velocity, and Concentration Contour

**Figure 4:**
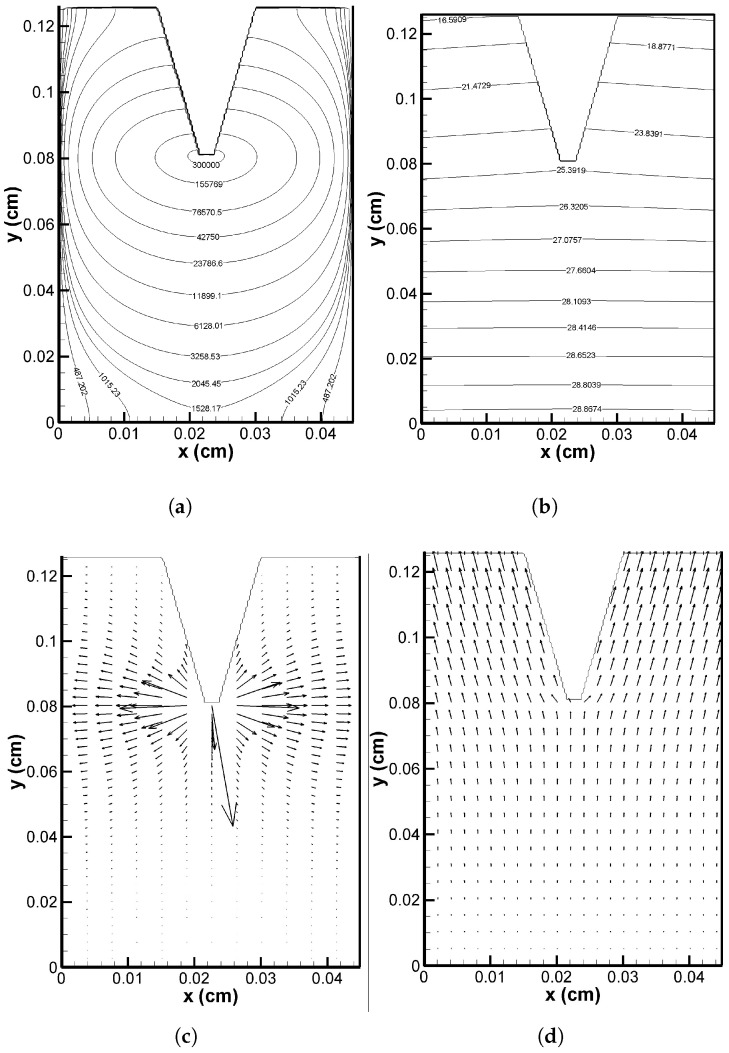
Pressure contour (in Pa unit) (**a**) during verapamil delivery; (**b**) after verapamil delivery. Velocity vector (**c**) during verapamil delivery (not to scale); (**d**) after verapamil delivery (not to scale). Application duration ($t_{a}$) = 4 h.

Figure 4a,b, respectively, show pressure contours for interstitial flow in the VS for two distinct scenarios: during verapamil delivery $\left(0\leq t\leq t_{a}\right)$ and after verapamil delivery $\left(t>t_{a}\right)$. It is essential to note that the injection velocity of verapamil delivered from the MN array caused a difference in the interstitial fluid pressure between the two cases. The vertical (transverse) interstitial velocity at the tip of the MN took on the injection velocity value during the delivery procedure, but it returned to zero when the delivery process ended. The maximum pressure was located at the tip of the MN, as seen in Figure 4a, and it decreased as one moved away from it. At the proximal ($\Gamma_{l},x=0$) and distal ($\Gamma_{r},x=L_{1}$) ends (cf. Figure 1), the mimimum pressure was detected. After MN application, a nearly uniform pressure could be seen throughout the domain (cf. Figure 4b). Therefore, it can be concluded that the MN array insertion significantly alters the interstitial fluid pressure across the VS. The velocity vectors in Figure 4c,d, respectively, illustrate the impact of pressure and local haemodynamics during and after the application of the MN array. During the application process, a greater velocity field was seen in the vicinity of the MN tip, and the velocity vector above a horizontal line through the tip was upward and was downward below it, owing to the combined effect of trans-epidermal water loss (Equation (1)) and effective injection velocity of verapamil (cf. Figure 4c). The velocity field significantly decreased after the application period and was always in the direction of $v_{i}^{y}$ (in this case, there was no average injection velocity ($u_{t}=0$), but still, trans-epidermal water loss occurred at the VS–SC interface; cf. Figure 4d). This makes sense, since upon administration, verapamil’s injection velocity at the MN tip increases the velocity field, which is thought to affect how verapamil disperses throughout the VS.

**Figure 5:**
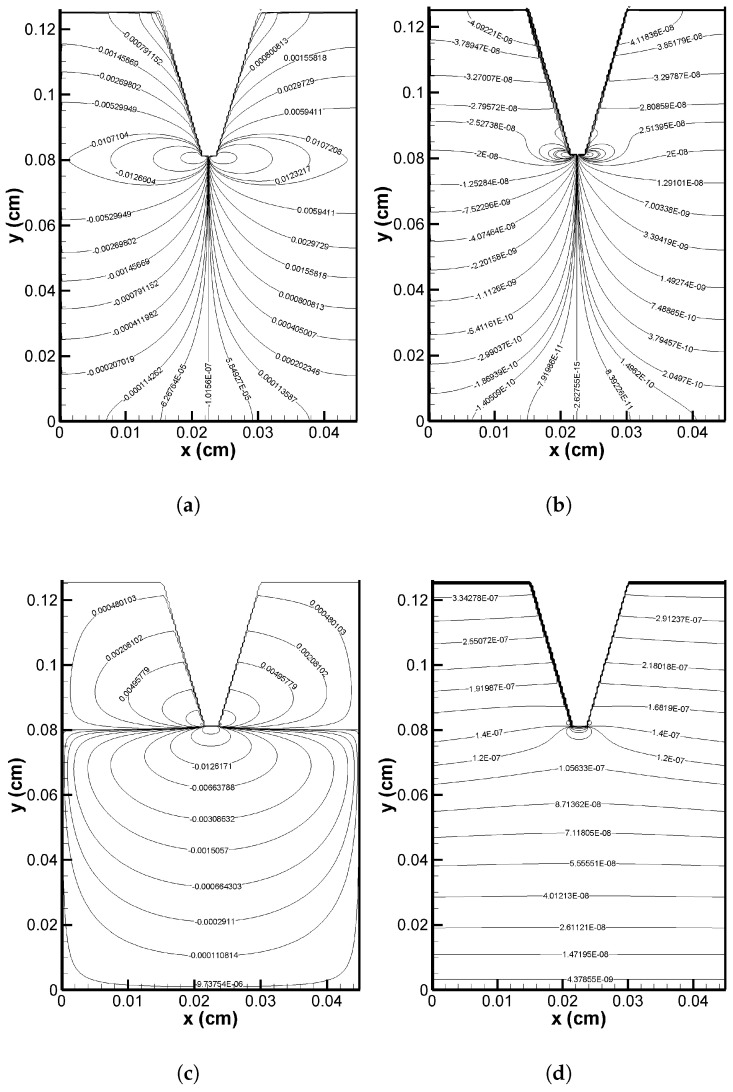
Axial velocity contour (in cm/s unit) (**a**) during verapamil delivery; (**b**) after verapamil delivery. Vertical velocity contour (in cm/s unit) (**c**) during verapamil delivery; (**d**) after verapamil delivery. Application duration ($t_{a}$) = 4 h.

The respective axial velocity contours of the interstitial fluid flowing through the porous VS during $\left(0\leq t\leq t_{a}\right)$ and after $\left(t>t_{a}\right)$ the application of MN array are shown in Figure 5a,b. Due to verapamil’s injection velocity throughout the verapamil delivery procedure, the axial velocity is always greater in absolute terms than after the application period. Specifically, the corresponding axial velocity changes sign when the interstitial fluid crosses the middle of the MN tip, implying the existence of a separatrix (a border dividing two different signs of axial velocity) inside the domain of investigation along which the axial velocity vanishes. Moreover, another interesting point is that the velocity was highest near the VS–SC interface following the cessation of drug delivery from the MN array and then decreased downward (cf. Figure 5b). At the same time, it was higher around the MN tip during the application duration (cf. Figure 5a). On the contrary, as illustrated in Figure 5c,d, respectively, the vertical velocity contours for $\left(0\leq t\leq t_{a}\right)$ and $\left(t>t_{a}\right)$ reveal some intriguing results. During the application time, a larger vertical velocity was seen near the tip (cf. Figure 5c), and the vertical velocity was positive above a horizontal line through the MN tip, which was negative below that line. This observation could be supported by the direction of vertical velocity, which was upward above the horizontal line across the MN tip and downward below the line. The vertical velocity reached its maximum at the VS–SC interface and remained positive across the domain after the application period. Since the domain is symmetric and there are symmetry boundary conditions at both the proximal and distal ends, the contours are symmetric in every instance.

**Figure 6:**
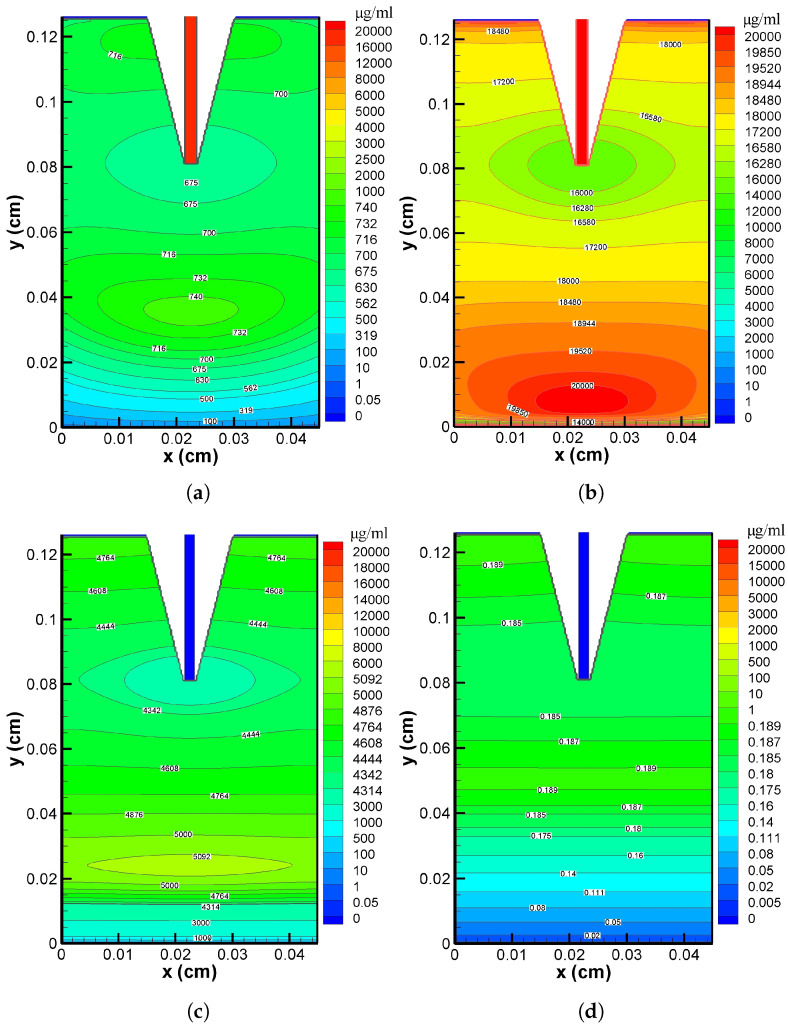
Concentration contour in VS at (**a**) 5 min, (**b**) 2 h, (**c**) 5 h, (**d**) 10 h. Application duration ($t_{a}$) = 4 h.

In Figure 6a–d, the verapamil dispersion over various time periods (5 min, 2 h, 5 h, and 10 h) is visually represented. Except for a small concentration near the skin–blood interface (y=0), the distribution patterns are less differential at t=5 min (cf. Figure 6a). This indicates that the concentration was nearly homogeneous throughout the two-dimensional domain. The pattern in Figure 6b illustrates the heterogeneous distribution at t=2 h. The concentration was at its highest at the VS–SC interface and decreased noticeably as one approached the needle tip; there was also a further rise towards the bottom of the domain and a subsequent decrease around the skin–blood interface area. A non-uniform distribution, similar to that shown in Figure 6b, but with a significantly smaller value (cf. Figure 6c), was observed at t=5 h when the MN stopped delivering verapamil. Finally, at t=10 h, a less differential verapamil distribution could be seen across the domain, except around y=0 (cf. Figure 6d). Based on the findings presented in Figure 6a–d, it can be inferred that the distribution patterns exhibit remarkable uniformity at the very beginning of the application time (5 min) and much beyond (10 h), and the domain was yet to be devoid of all verapamil molecules at t=10 h.

### 3.3. Impact of Aspect Ratio (AR)

The aspect ratio (AR) of MN geometry has been defined in many ways depending on the study. The length-to-width ratio, for instance, emphasises the needle’s overall geometry and mechanical properties [95]; the ratio of the base diameter to the tip diameter emphasises penetration efficiency [96]; and the ratio of MN length to tip diameter [97] are some examples of how researchers define the AR. Each definition considers different priorities, such as safety, penetration, or mechanical strength. The definition of AR in our study is the ratio of the length (L) of the MN to its base diameter (R); this can be written as AR = $\frac{L}{R}$. The baseline value of AR = 3 in this investigation was set as L=450 μm and R=150 μm. There are two approaches to vary the AR:

Case-I involves changing *L* while maintaining *R* fixed at its baseline value (R=150 μm).

Case-II involves changing *R* while maintaining *L* fixed at its baseline value (L=450 μm).

#### 3.3.1. Case I

**Figure 7:**
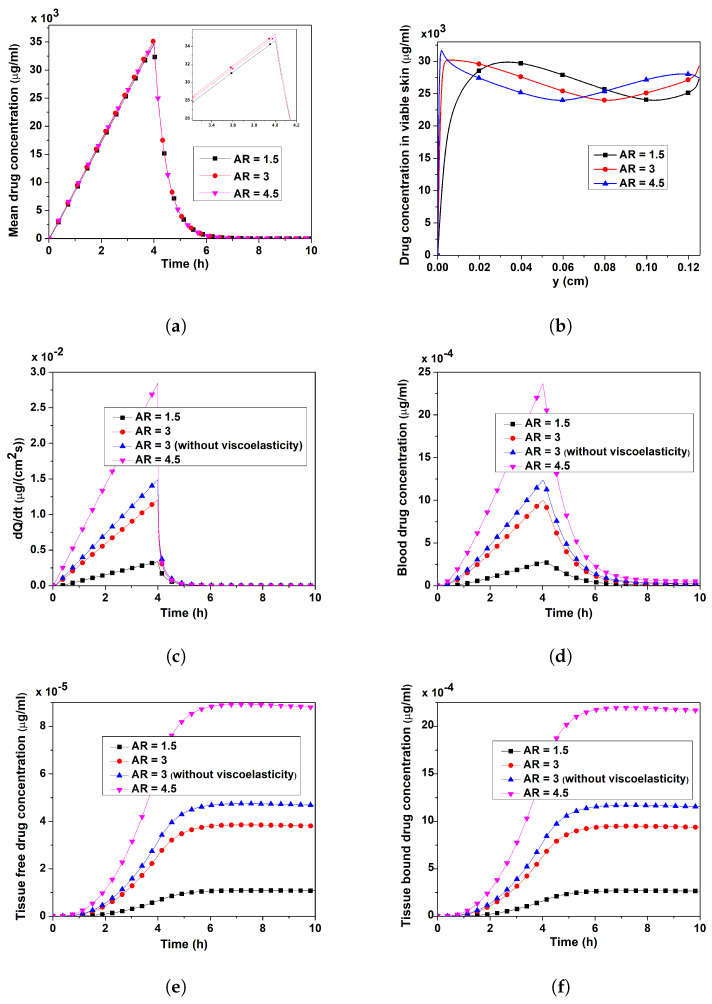
Case I: Impact of AR (AR = $\frac{L}{R}$; *R* is fixed, but *L* varies) on (**a**) mean verapamil concentration in VS, (**b**) vertical profile of verapamil concentration in the VS at x=0.0135 cm, (**c**) rate of verapamil permeation into blood compartment, (**d**) blood concentration, (**e**) tissue-free verapamil concentration, (**f**) tissue-bound verapamil concentration. Application duration ($t_{a}$) = 4 h.

Figure 7a,c–f show the temporal variations of verapamil’s mean concentration in VS, the rate of verapamil permeation, the blood concentration, and the concentrations of both bound and free verapamil for various ARs (when *R* is fixed but *L* varies). The mean verapamil concentration in the skin rose with increasing AR from its lowest value and then progressively decreased after the MN array application ($t_{a}\;=\;4$ h). Moreover, when the AR increased from 1.5 to 3, the mean concentration of verapamil rose. The most noteworthy finding, though, is that the mean concentration decreased more at an AR of 4.5 than it did at an AR of 3 (zoomed inset). This does suggest that a greater mean concentration in the VS is not always associated with a longer MN length. Figure 7b shows how the AR influences the *y*–profile of verapamil concentration in the VS when *x* = 0.0135 cm at t=2 h, which helps to explain the mean concentration’s undulating nature. Furthermore, the concentration around the bottom end (VS–blood compartment interface, y=0) increased as the AR increased, but this was not given for the remaining vertical height, resulting in an undulating mean concentration.

The temporal change in the verapamil permeation rate ($\frac{dQ}{dt}$) into the blood compartment and blood concentration ($C_{b}$) for different ARs are shown in Figure 7c,d, respectively. Because of an increase in concentration in skin with increasing AR near y=0 (cf. Figure 7b), the rate of verapamil permeation into the blood compartment increased with increasing AR (=1.5, 3, 4.5), and eventually reduced to zero beyond 4 h (cf. Figure 7c). By comparing the curves for AR = 3, it can be said that the MN array’s transdermal verapamil delivery is resistively affected by the skin’s viscoelasticity. A steep decline in the permeation rate was noted for higher values of AR. A higher AR took longer time to remove all verapamil molecules from the blood compartment, and the verapamil concentration in the blood compartment increased as the AR grows (cf. Figure 7d). Additionally, the blood concentration was decreased by the viscoelasticity of the VS. The absorption of verapamil from the blood compartment into the tissue and its subsequent binding, which the AR impact does, are crucial factors to consider when evaluating the efficacy of the transdermal verapamil delivery system. Remarkably, the concentration of free verapamil in tissue reached a maximum long after the MN array ceased to deliver drug, and then it gradually decreased as a result of verapamil entering the blood compartment from the tissue and binding to specific receptors (cf. Figure 7e). The tissue exhibited comparable behaviour when verapamil was bound (cf. Figure 7f). Bound verapamil eventually diminished because it transformed into an unbound (free) state over time. It is evident from Figure 7e,f that the curve for the non-viscoelastic skin shows a significant increase compared to that of the viscoelastic skin at the same aspect ratio. Comparing all the figures, one may conclude that the increasing AR amplifies the concentrations of all forms in the blood and tissue compartments owing to the increasing permeation rate, even though the mean concentration in the VS is of an undulating nature, and the viscoelasticity of the skin acts as a resistive barrier for MN-based verapamil delivery.

#### 3.3.2. Case II

**Figure 8:**
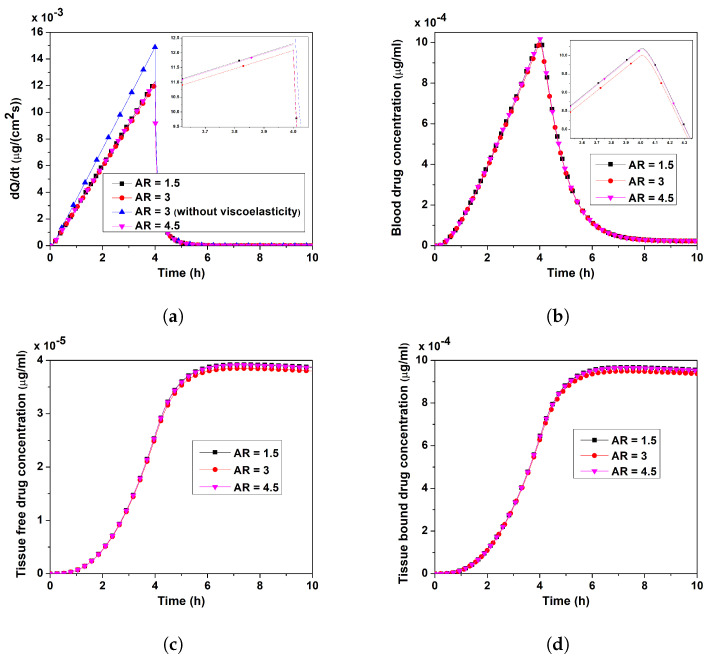
Case II: Impact of AR (AR = $\frac{L}{R}$; *L* fixed, but *R* varies) on (**a**) rate of verapamil permeation into blood compartment, (**b**) blood concentration, (**c**) tissue-free verapamil concentration, (**d**) tissue-bound verapamil concentration. Application duration ($t_{a}$) = 4 h.

Unlike Case I, in this scenario (Case II), a rise in AR inevitably resulted in a decrease in the base diameter (*R*), ultimately increasing the area of the computational domain. For different ARs, Figure 8a–d show how the verapamil permeation rate and concentrations of verapamil in the blood and tissue compartments changed momentarily when the MN’s length *L* was fixed, but its base diameter *R* varied. Following the application of the MN, the rate of verapamil permeation and blood concentration climbed from zero to a peak value for all AR values (=1.0, 3, 4.5) examined and then progressively declined (cf. Figure 8a,b). Figure 8c,d depict the temporal changes of both free and bound verapamil in the tissue compartment for various ARs, respectively. After peaking, the concentrations of both verapamil forms gradually started to decrease. In contrast to Figure 7c–f, as seen in Figure 8a, the permeation rate decreased from AR = 1.5 to 3 and then increased from AR = 3 to 4.5. A similar trend can be seen in the blood and tissue compartments. The reversible binding of free verapamil with specific receptors within the tissue compartment was the cause of the diminishing profiles for both free and bound verapamil after t∼7 h (cf. Figure 8c,d). Based on a comparison of Figure 7 and Figure 8, it can be inferred that alterations in the AR, as in Case I, perturbed the MN-based drug delivery more than in Case II.

### 3.4. Impact of Effective Injection Velocity ($u_{0}$)

**Figure 9:**
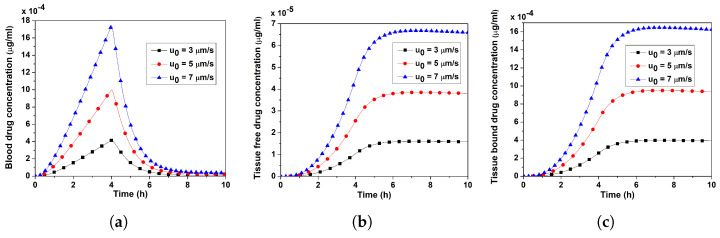
Impact of the effective injection velocity ($u_{0}$) on (**a**) blood concentration, (**b**) tissue-free verapamil concentration, (**c**) tissue-bound verapamil concentration. Application duration ($t_{a}$) = 4 h.

**Figure 10:**
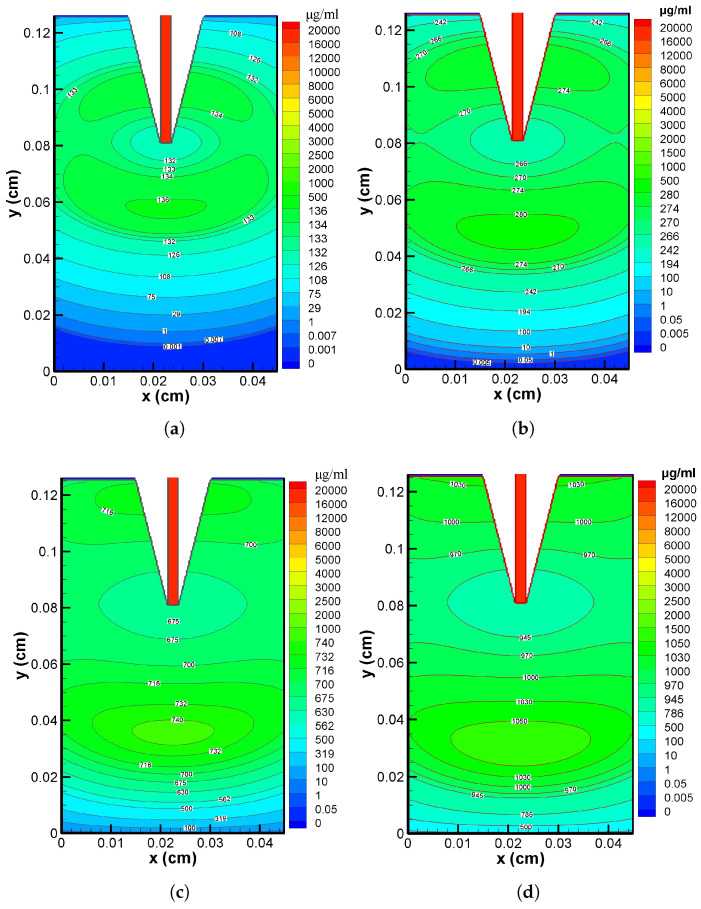
Concentration contour in the VS at t=5 min (**a**) $u_{0}$ = 1 μm/s, (**b**) $u_{0}$ = 2 μm/s, (**c**) $u_{0}$ = 5 μm/s, (**d**) $u_{0}$ = 7 μm/s. Application duration ($t_{a}$) = 4 h.

It may be recalled that the effective injection velocity ($u_{0}$) and the average injection velocity of a single MN ($u_{t}$) are related by $u_{t}\left(=\frac{u_{0}S}{nS_{t}}\right)$ (cf. Equation (25)). The effects of the effective injection velocity (=3, 5, 7 μm/s) on the verapamil concentration in the blood and tissue compartments are depicted in Figure 9a–c. A higher effective injection velocity would increase the verapamil concentration over the blood and tissue compartments due to a higher permeation of verapamil into the blood compartment (cf. Figure 10). In other words, the concentrations in blood and tissue compartments are proportional to the value of $u_{0}$. Visual representations of the concentration contour in the VS for various $u_{0}(=1,2,3,7$ μm/s) are depicted in Figure 10a–d. The observations reveal that the greater the $u_{0}$, the higher the verapamil concentration near y=0, increasing the flux to the blood compartment. As a result, raising the effective injection velocity can improve MN-based drug delivery; however, it should be noted that doing so necessitates applying a lot of pressure to the MN. The MN may break if the force applied exceeds its breaking strength. A seminal study highlighted determining two crucial parameters: (i) the force needed to embed MN in living skin and (ii) the force at which MN may be subjected before breaking [29]. As long as the MN can withstand the external force, the effective injection velocity can be considered a controlling parameter to achieve the therapeutic efficacy of MN-based verapamil delivery.

### 3.5. Impact of Tip Diameter ($d_{tip}$) of the MN

The change in tip diameter can lead to a change in the area of the MN tip ($S_{t}$) (=$\frac{\pi d_{tip}^{2}}{4}$), which in turn can vary the average injection velocity of a single MN ($u_{t}$), according to the relation $u_{t}\;=\;\frac{u_{0}S}{nS_{t}}$ (Equation (25)). Yet, keeping the effective injection velocity ($u_{0}$) fixed at its baseline value, we have looked into two scenarios in this investigation, specifically including the following:

Case I: Increasing $d_{tip}$ would result in a decrease in $u_{t}$ via increasing $S_{t}$. Here, there is no change in verapamil flux from the MN tip.

Case II: While $u_{t}$ would remain constant at its baseline value, an increase in $d_{tip}$ would increase in $S_{t}$ and a rise in verapamil flux from the MN tip.

#### 3.5.1. Case I

**Figure 11:**
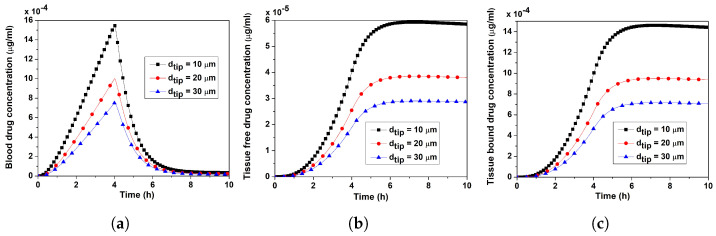
Case I: Impact of the tip diameter ($d_{tip}$) on (**a**) blood concentration, (**b**) tissue-free verapamil concentration, (**c**) tissue-bound verapamil concentration. Application duration ($t_{a}$) = 4 h.

Figure 11a–c depict the temporal variations of blood concentration and tissue compartment’s concentrations (free and bound). In Case I, $u_{t}$ decreased four and nine times, respectively, in response to a two- and three-fold increase in $d_{tip}$. Nevertheless, during the application, the verapamil flux from the MN tip did not change (Equation (25)). Because of a faster average injection velocity of a single MN ($u_{t}$), the concentrations in the blood and tissue compartments were all-time larger for smaller $d_{tip}$(=10 μm). When $d_{tip}$ rose, the concentration fell sharply (i.e., $u_{t}$ lowered).

#### 3.5.2. Case II

**Figure 12:**
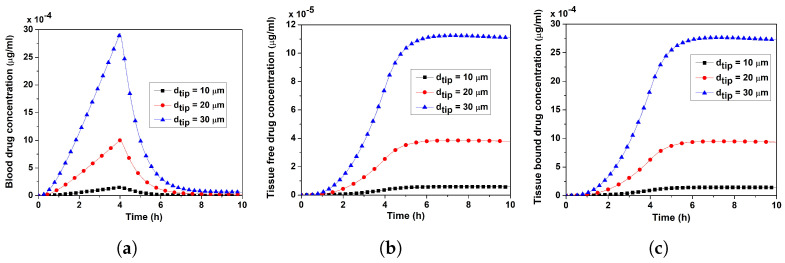
Case II: Impact of the tip diameter ($d_{tip}$) on (**a**) blood concentration, (**b**) tissue-free verapamil concentration, (**c**) tissue-bound verapamil concentration. Fixed average injection velocity ($u_{t}$). Application duration ($t_{a}$) = 4 h.

On the contrary, in Case II, with $u_{t}$ held constant at its baseline value, the increasing $d_{tip}$ enhanced the tip’s area, ultimately leading to an increase in the verapamil flux from the MN tip (Figure 12a–c; Equation (25)). At this instance, when $d_{tip}$ increased, so too did the concentrations in the blood and tissue compartments. Therefore, if the MN is strong enough to withstand the force required to insert a wider tip into the skin—the tip diameter in the case of MN-based verapamil administration—similar to effective injection velocity, it may function as a regulating parameter for the effectiveness and efficacy of the therapy.

## 4. Discussion

In comparison to oral and hypodermic needle delivery, the MN-based TDD is better because of skin’s protective, inflammatory, and immunological properties. We still have a lot to learn about the way drugs are delivered using MN as well as how well they work when the skin is considered as a viscoelastic medium. Clinical studies have not entirely demonstrated that they produce a long-lasting, persistent benefit. Our research suggests that viscoelasticity of skin, the presence of ISF and the retention of drug in the tissue have a substantial impact on MN-based delivery. These trials can be guided by animal models and pre-clinical research.

In this study, a hollow microneedle-mediated verapamil delivery model with a monolayer viscoelastic skin and a two-compartment body has been taken into consideration. Verapamil released from the HMN tip diffused in the two-dimensional domain with interstitial flow and was absorbed in the blood compartment. The amount of fluid lost through the SC and to the lymphatic system has been appropriately considered. In addition to systemic clearance from the blood, there was a reversible uptake kinetics dynamic observed between the blood and tissue compartments. Consideration has been given to reversible and saturable binding kinetics, since verapamil is thought to bind to the α-adrenergic receptors found in the tissue [60,61]. The skin becomes viscoelastic due to MN insertion, which causes the fluid to diffuse according to Fickian and non-Fickian diffusion laws [54]. The Marker and Cell [93] and Immersed Boundary Methods [65] were used to numerically solve the governing equations that reflect verapamil transport in the two-dimensional viscoelastic irregular domain, the blood (plasma) concentration, and the free and bound verapamil concentrations in the tissue compartment. This study looked into how MN-based verapamil delivery affects interstitial pressure and velocity. In this investigation, it was impossible to rule out the effects of the aspect ratio (AR), tip diameter, and effective injection velocity on the transient concentrations. As far as the authors are aware, the binding of verapamil in the tissue compartment using MN-based verapamil delivery in two-dimensional viscoelastic skin has not been considered in any earlier investigations.

The interstitial fluid pressure and the velocity field—which are crucial for the convective transport of verapamil in the viscoelastic VS—will be greatly impacted by the insertion of MN arrays (cf. Figure 4 and Figure 5). According to Figure 6, the concentration of verapamil shows various patterns both during and after the application period, which indicate the transient behaviour of verapamil transport in the domain under consideration. The results predict that a longer MN length is not always correlated with a higher mean concentration in the VS. However, as in Case I (cf. Figure 7), increasing the AR increased the concentrations in the blood and tissue compartment, but as in Case II (cf. Figure 8), increasing the AR exhibited oscillatory behaviour in the blood and tissue concentrations. The findings of the simulations indicate that the concentrations in the skin and compartments are more perturbed by the AR, as described in Case I. Since the concentration of bound verapamil in the tissue is larger at higher injection velocities, the effective injection velocity can be considered a regulating parameter for the therapeutic efficacy of the MN-based verapamil delivery (cf. Figure 9). An alteration in the tip diameter modified the average injection velocity of a single MN while maintaining an unaltered total verapamil flux (Case I; Equation (25)). Blood and tissue concentrations increased with smaller tip diameter (greater injection velocity; cf. Figure 11), but they decreased with decreased verapamil flux (Case II; cf. Figure 12).

After analysing every simulation result, it can be concluded that various factors need to be optimised to comprehend MN-mediated verapamil delivery’s complete success. Our study undoubtedly opens the door to a more profound understanding of MN-based TDD. Yet, precise confirmation through animal or benchtop experiments may enable more accurate estimation. The findings of this investigation could be employed to corroborate these experimental outcomes. 

## Figures and Tables

**Table 1:** Pharmacokinetics variables used for analysing the concentration of verapamil delivered from MN array.

Parameters	Values	References
x− length of the VS domain: $L_{1}$ (μm)	450	our study
Density of verapamil: $\rho_{d}$ (g cm^−3^)	1.1	our study
Volume of skin: $V_{skin}$ (cm^3^)	0.1265	our study
Surface area of the MN array: *S* (cm^−2^)	1	[31]
Distance to VS-blood compartment interface from the top of SC: *h* (μm)	1265	[78]
Thickness of the SC: AB (μm)	15	[31]
MN length: *L* (μm)	450	[31]
Effective injection velocity of verapamil: $u_{0}$ (μm s^−1^)	5	[84]
Diameter of the MN tip: $d_{tip}$ (μm)	20	[31]
Number of needles in an MN array: n	400	[31]
Base diameter of MN: *R* (μm)	150	[31]
Aspect ratio (AR = $\frac{L}{R}$)	3	[31]
Duration of MN application: $t_{a}$ (h)	4	[8]
Stress-driven diffusion coefficient: $D_{v}$ (μg/(s cm Pa))	$10^{-14}$	[54]
Metabolic rate constant of verapamil in VS: $k_{m}$ (s^−1^)	$5.61\times 10^{-4}$	[37]
Young modulus of the free spring: $E_{0}$ (Pa)	$5.22\times 10^{5}$	[50]
Young modulus of the Maxwell fluid: $E_{1}$ (Pa)	$8.05\times 10^{5}$	[50]
Viscosity of Maxwell fluid: μ (Pa s)	6.5$\times 10^{5}$	[54]
Dimensional positive constant: α (mL/μg)	1	[54]
Darcy permeability of the interstitium: *k* (cm^2^)	$1.0\times 10^{-12}$	[81]
Porosity of VS: ϕ	0.2	[85,86]
Tortuosity of VS: $\tau_{s}$	1.2	[87]
Osmotic pressure of blood in VS: $\pi_{bl}$ (Pa)	2670	[80]
Osmotic pressure of interstitial fluid in VS: $\pi_{i}$ (Pa)	1330	[80]
Osmotic reflection coefficient for blood proteins in VS: $\sigma_{T}$	0.91	[80]
Hydraulic conductivity of the blood vessel wall: $L_{bl}$ (m/Pa/s)	$2.7\times 10^{-12}$	[80]
Intracapillary pressure: $p_{bl}\left(Pa\right)$	2080	[80]
Capillary surface area per tissue volume: $\frac{S_{bl}}{V_{tis}}\left(m^{-1}\right)$	$6.0\times 10^{3}$	[88]
Transport rate of interstitial fluid to lymphatics: $L_{ly}\frac{S_{ly}}{V_{tis}}\left(Pa^{-1}s^{-1}\right)$	$4.2\times 10^{-7}$	[80]
Intra-lymphatic pressure: $p_{ly}$ (Pa)	0	[80]
Ambient relative humidity: RH	0.7	[82]
Density of interstitial fluid: $\rho_{f}\;\;$ (g cm^−3^)	1.0	[89]
Viscosity of the fluid: $\mu_{f}$ (Pa s)	$7.8\times 10^{-4}$	[90]
Diffusion coefficient in VS: $D_{vs}$ (cm^2^ s^−1^)	$9.75\times 10^{-8}$	[8]
Volume of distribution in blood: $v_{b}$ (mL)	26,300	[73]
Volume of distribution in tissue: $v_{t}\left(mL\right)$	51,800	[73]
Elimination rate constant: $k_{e}$ (s^−1^)	$1.58\times 10^{-4}$	[73]
Transfer rate constant from blood to tissue: $k_{12}$ (s^−1^)	$2.19\times 10^{-4}$	[73]
Transfer rate constant from tissue to blood: $k_{21}$ (s^−1^)	$1.11\times 10^{-4}$	[73]
Initial binding site concentration: $B_{M}$ (μg/mL)	64,064× 10^−5^	[61]
Association (binding-on) rate constant: $k_{a}$ (s^−1^) (μg/mL)^−1^	3.74	[61]
Dissociation (binding-off) rate constant: $k_{d}$ (s^−1^)	0.097	[61]

## Data Availability

The data are contained within the article.
